# Discovery of NFκB2-Coordinated Dual Regulation of Mitochondrial and Nuclear Genomes Leads to an Effective Therapy for Acute Myeloid Leukemia

**DOI:** 10.3390/ijms25158532

**Published:** 2024-08-05

**Authors:** Yi Xu, David J. Baylink, Jeffrey Xiao, Lily Tran, Vinh Nguyen, Brandon Park, Ismael Valladares, Scott Lee, Kevin Codorniz, Laren Tan, Chien-Shing Chen, Hisham Abdel-Azim, Mark E. Reeves, Hamid Mirshahidi, Guido Marcucci, Huynh Cao

**Affiliations:** 1 Division of Hematology and Oncology, Department of Medicine, School of Medicine, Loma Linda University, Loma Linda, CA 92354, USA; cschen@llu.edu (C.-S.C.);; 2 Division Regenerative Medicine, Department of Medicine, School of Medicine, Loma Linda University, Loma Linda, CA 92354, USA; 3 Cancer Center, Loma Linda University, Loma Linda, CA 92354, USA; 4 Division of Endocrinology, Diabetes & Metabolism, Department of Medicine, School of Medicine, Loma Linda University, Loma Linda, CA 92354, USA; 5 Division of Pulmonary, Critical Care, Hyperbaric and Sleep Medicine, Department of Medicine, School of Medicine, Loma Linda University, Loma Linda, CA 92354, USA; 6 Division of Transplant and Cell Therapy, Loma Linda University Cancer Center, Loma Linda, CA 92354, USA; 7 Division of Hematology and Oncology, Department of Pediatrics, Loma Linda University, Loma Linda, CA 92354, USA; 8 Department of Hematological Malignancies Translational Science, Gehr Family Center for Leukemia Research, City of Hope Medical Center and Beckman Research Institute, Duarte, CA 91010, USA

**Keywords:** AML, NFκB2, FLT3, gilteritinib, mitochondrion, TFAM, NRF1, ATP, metabolism

## Abstract

Acute myeloid leukemia (AML) has a poor survival rate for both pediatric and adult patients due to its frequent relapse. To elucidate the bioenergetic principle underlying AML relapse, we investigated the transcriptional regulation of mitochondrial–nuclear dual genomes responsible for metabolic plasticity in treatment-resistant blasts. Both the gain and loss of function results demonstrated that NFκB2, a noncanonical transcription factor (TF) of the NFκB (nuclear factor kappa-light-chain-enhancer of activated B cells) family, can control the expression of TFAM (mitochondrial transcription factor A), which is known to be essential for metabolic biogenesis. Furthermore, genetic tracking and promoter assays revealed that NFκB2 is in the mitochondria and can bind the specific “TTGGGGGGTG” region of the regulatory D-loop domain to activate the light-strand promoter (LSP) and heavy-strand promoter 1 (HSP1), promoters of the mitochondrial genome. Based on our discovery of NFκB2′s novel function of regulating mitochondrial–nuclear dual genomes, we explored a novel triplet therapy including inhibitors of NFκB2, tyrosine kinase, and mitochondrial ATP synthase that effectively eliminated primary AML blasts with mutations of the FMS-related receptor tyrosine kinase 3 (*FLT3*) and displayed minimum toxicity to control cells ex vivo. As such, effective treatments for AML must include strong inhibitory actions on the dual genomes mediating metabolic plasticity to improve leukemia prognosis.

## 1. Introduction

Acute myeloid leukemia (AML) continues to be a challenge to treat since the introduction of cytarabine/daunorubicin (7 + 3) in 1973 (50 years ago) [1,2]. Among diverse oncogenic factors, mutations of the FMS-related receptor tyrosine kinase 3 (*FLT3*) are expressed in ~35% of AML patients (*FLT3*-mut AML), who respond poorly to salvage chemotherapies [3]. Even with the advent of new FLT3 inhibitors, such as tyrosine kinase inhibitors (TKIs), the median overall survival in relapsed/refractory AML is less than one year [4,5]. In this regard, a combination of venetoclax, a B-cell lymphoma 2 (BCL-2)-inhibitor, and TKI treatment has been explored in vitro and in vivo, displaying synergistically improved treatment efficacy [6]. Also, the combination regimen of venetoclax-gilteritinib is in clinical trials for *FLT3*-mut AML patients; however, response rate, resistance, and relapse still represent major clinical challenges [7].

In our recent effort to determine the refractory mechanism, we discovered that transient CD44 + pBAD + (phosphorylated BCL2 associated agonist of cell death +) blasts underwent complex intrinsic homeostasis to elude TKI-treatment [8,9]. Therefore, developing a fundamental pathway-driven targeted therapy that can treat heterogeneous AML patients and overcome their divergent refractory mechanisms is an unmet need and is critical to improving AML prognosis [10,11,12,13].

Leukemia cells (blasts) can proliferate at a rate that is 20 times normal, demanding equivalently increased metabolism. Therefore, a logical therapeutic target should correspondingly reduce the mitochondrial energy conversion chain. However, blasts can adapt their cellular metabolism to meet the higher demands of the energy and metabolites necessary to elude treatment and drive AML progression [8,14,15,16]. Moreover, uncontrolled blasts take advantage of their microenvironment by depriving nutrients from the surrounding hematopoietic environment, leading to immunodeficiency and failure of bone marrow regeneration [17]. In this regard, we reasoned that targeting the adaptive capability of energy conversion and the metabolism of blasts [18] could be a therapeutic priority to actively prevent (or at least robustly delay [19]) leukemia relapse so that the long-term survival and quality of life will be improved for AML patients [20,21]. Thus, we began our search for a novel target within the AML mitochondrial system and beyond.

For background, the mitochondrion is a hub for many fundamental processes from adenosine triphosphate (ATP) energy production to signaling for stress response and apoptosis, and it plays critical roles in a myriad of human diseases and ageing [22,23,24]. Notably, the mitochondrion possesses its own genome (mtDNA) that serves to maintain the integrity of its membrane potentials and organelle structures, which are essential for electron/protein transport and the oxidative phosphorylation (OXPHOS) system required for ATP and metabolite production [25,26,27]. MtDNA is composed of a closed circular DNA genome that encodes thirteen core proteins involved in the five OXPHOS complexes to produce ATP, including seven subunits of the NADH dehydrogenase (ND: ND1, 2, 3, 4, 4L, 5, and 6) (Complex I), the cytochrome *b* (CytB) (Complex III), three subunits of the cytochrome *c* oxidase (COX) (Complex IV), and two subunits of ATP synthase (ATP6 and ATP8) (Complex V) [28]. Therefore, perturbed mitochondrial genome and bioenergetic dysfunction have been correlated with oncogenesis and cancer progression [29,30].

Importantly, the vast majority (99%) of the ~1500 structural and functional proteins for the OXPHOS system are encoded by the nuclear genome (nDNA) and imported into the mitochondria [31]. For example, human mitochondrial transcription is governed by a nuclear-encoded single-subunit RNA polymerase (POLRMT) that is assisted by two nuclear-encoded transcription initiation factors—mitochondrial transcription factor A (TFAM) and mitochondrial transcription factor B2 (TFB2M)—to drive transcription of mtDNA [32]. Therefore, it would seem that the mitochondria need to communicate with the nuclei through anterograde and retrograde signaling processes to undergo dual genomic reprogramming that will maintain organelle homeostasis and carry out the metabolic adaptations necessary to support cell survival [33,34].

Unfortunately, a long-standing question for human biology is how mitochondrial–nuclear genomes coordinate to encode proteins that are essential for the assembly and maintenance of a functional OXPHOS system required for energy conversion under both physiologic and pathologic states [35,36,37]. Among the potential regulators of the communication and genetic changes between dual genomes, transcription factors (TFs) are most likely to be involved in mitochondrial protein homeostasis [38,39]. TFs are a group of proteins that bind to a specific DNA sequence to control the rate of transcribing genetic information from DNA to messenger RNA and are thus essential for the regulation of gene expression in all organisms [40]. Three classes of TFs are known to be important in human cancer, including (1) the NF-kappaB and activator protein-1 (AP-1) families, (2) the signal transducer and activators of transcription (STAT) family, and (3) the steroid receptors [41]. Due to their important roles in intercellular signaling and cell cycle, TFs could be therapeutic targets, leading to approximately 10% of currently prescribed drugs directly targeting the nuclear receptor class of TFs [42,43].

Although the mechanism of basal transcription machinery essential for mtDNA has been potentially defined [44], none of the relevant published TFs (e.g., TFAM, TFB2M, etc.) is known to regulate the dual genomes required for metabolic plasticity in AML blasts. In this regard, we hypothesize that there are other yet-to-be-determined pivotal TFs that support metabolic adaptation in blast mitochondria, especially under the pathologic conditions and antileukemia treatments. The identification of such TFs and the characterization of their interaction networks could uncover effective targets to terminate uncontrolled blast proliferation and prevent AML relapse beyond targeting metabolism alone [45].

Among three cancer-related TF families mentioned above, we selected the Nuclear factor kappa-light-chain-enhancer of activated B cells (NF-kappaB, NFκB, or NFKB) for further studies. The NFκB is a family of TF complexes that controls DNA transcription, cytokine production, and cell survival in response to external stimuli through the activation of canonical and noncanonical NFκB pathways, which have different cell surface receptors, cytoplasmic adaptors, and NFκB dimers [46]. In contrast to the extensively studied canonical signaling pathway, the involvement of the noncanonical NFκB2 pathway in human cancers is less defined [47]. Previously, we discovered that TKI-activated NFκB2 is the key regulator of C-X-C motif chemokine ligand (CXCL)/CXC receptor 2 (CXCR2)-mediated inflammatory pathways in promoting the survival and relapse of *FLT3*-mut AML blasts ex vivo [9]. Notably, the standard AML treatment, daunorubicin, was also recently found to upregulate NFκB2 activities in blasts [48]. Therefore, the inhibition of NFκB2-mediated survival pathways could potentially overcome the TKI resistance of AML patients [49].

In this work, to identify the novel target responsible for mitochondrial biogenesis and metabolic reprogramming in refractory blasts, we designed serial in vitro experiments utilizing multiomic approaches to investigate the fundamental mechanism underlying the coordinated transcriptional regulation of mitochondrial–nuclear dual genomes after their exposure to targeted treatments. Importantly, based on our mechanistic findings, we developed a novel triplet therapy to effectively treat AML and overcome the resistance phenotype.

## 2. Results

### 2.1. Oligomycin-Inhibiting ATP Synthase (Complex V) Can Sensitize Blasts to TKI-Treatment; However, Refractory Blasts Underwent the Genomic Reprogramming Required for Metabolic Plasticity

**Figure 1:**
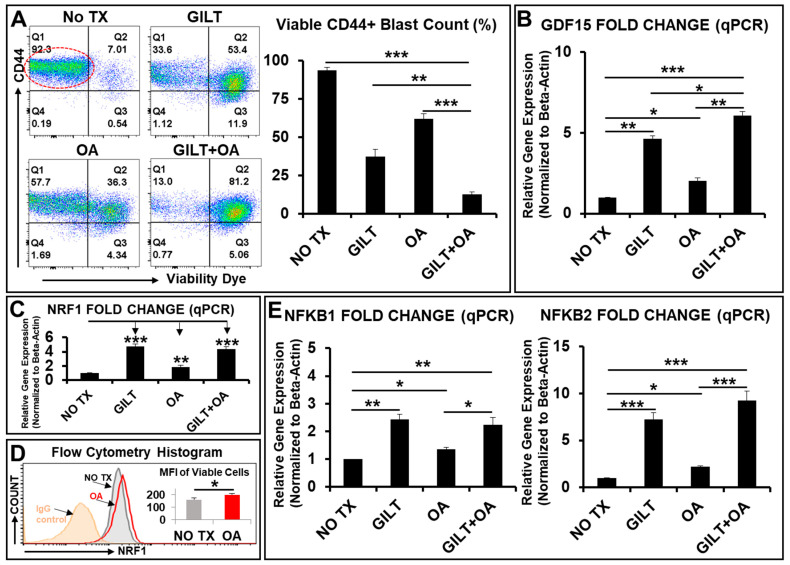
Mitochondria (ATP synthase, Complex V)-targeted therapy sensitized blasts to TKI treatments but nuclear genomes of blasts underwent reprogramming to survive. (N = 3). (**A**) Representative FC plots of different experimental groups with NO TX, 80 nM gilteritinib (GILT), 100 nM oligomycin (OA), and GILT + OA on viability dye and CD44 expression; Red circle indicates CD44+/viability dye- (negative) viable blasts; Right table: Cumulative percentage data of viable CD44+ cells in different treatment groups; (**B**) Gene expression of *GDF15*, a biomarker for mitochondrial diseases or oxidative stress, was analyzed by qPCR. Data of mRNA expressions show the fold change (normalized to *β-actin*) of *GDF15* in different treatment groups; (**C**) Gene expression of *NRF1* (nuclear respiratory factor 1), a key transcription regulator of mitochondrial biogenesis, was analyzed by qPCR. Data of mRNA expressions show the fold change (normalized to *β-actin*) of *NRF1* in different treatment groups; (**D**) Representative FC histograms showing expression of NRF1 in viable MV4-11 cells that had IgG-staining control (orange plot line); NO TX (gray plot line); 100 nM oligomycin (OA)-treated experimental groups (red plot line); Right table: Cumulative MFI (mean fluorescence intensity) data of NRF1 + viable cells of different treatment groups; (**E**) Gene expressions of *NFκB1* and *NFκB2*, transcription factors that rapidly respond to cellular stimuli, were analyzed by qPCR. Data of mRNA expressions show the fold change (normalized to *β-actin*) of *NFκB1* and *NFκB2* in different treatment groups; Where applicable, data are means ± SEM. * *p* < 0.05, ** *p* < 0.01, *** *p* < 0.005.

In our recent studies to determine the refractory mechanisms of AML, we discovered that transient CD44 + pBAD + blasts underwent complex intrinsic homeostasis to elude TKI-treatment [8,9]. To improve upon the TKI treatment, we explored a combination approach to target mitochondrial metabolism to treat AML blasts (Figure 1). Oligomycin (OA) is an antifungal antibiotic and can effectively inhibit ATP synthase (complex V) of the OXPHOS system, leading to the disruption of tumor progression [50]. Then, we performed flow cytometry (FC) to analyze the viability dye (positive indicates dying or dead cells) and CD44 expression, as well as an AML blast marker to determine the therapeutic efficacy of the combination in vitro. FC plots showed that the NO TX group had a viable blast count (CD44+/viability dye negative, indicated by red circle) of 92.3%, gilteritinib (GILT) had a viable blast count of 33.6%, OA had a viable blast count of 57.7%, and GILT + OA had a viable blast count of 13% (Figure 1A), demonstrating the enhanced antileukemia effect of the GILT + OA combination.

Next, to determine the gene expression changes in treated blasts, we performed qPCR analyses of Growth Differentiation Factor 15 (*GDF15*), which is a biomarker for mitochondrial diseases or oxidative stress [51,52], as well as of a group of nuclear TFs responsible for blast survival and metabolic adaptation. *GDF15* was significantly increased in expression for all treatments compared to NO TX, but GILT- and GILT + OA-treated cells had even higher *GDF15* expressions than OA treatment alone (4.6-fold and 6-fold increase compared to 2-fold increase) (Figure 1B). Notably, qPCR analyses showed a significant increased expression (by more than 4.7-fold for GILT and 4.4-fold for GILT + OA compared to NO TX) of nuclear respiratory factor 1 (*NRF1*), which is a master regulator of mitochondrial biogenesis by binding *TFAM* and *TFB2M* promoters, and mediating mitochondrial–nuclear interactions (Figure 1C) [53,54]. Furthermore, FC analyses revealed that OA treatment could significantly increase the protein expression of NRF1 (MFI: ~200) in viable blasts when compared to NO TX (MFI: ~162) (Figure 1D). Finally, consistent with our previous report [9], the *NFκB* family also significantly increased in gene expression in all treatment groups (Figure 1E). GILT- and GILT + OA-treated cells had the highest increases in *NFκB2* expression (7.2-fold increase and 9.2-fold increase, respectively) compared to NO TX, which were even higher than the respective *NFκB1* expression increases (2.4-fold and 2.2-fold in GILT and GILT + OA groups) compared to NO TX (Figure 1E).

In summary, our data demonstrate that the supplementation of OA that targets mitochondrial ATP synthase could significantly improve GILT’s therapeutic effect; however, both single and combination treatments activated robust compensatory responses of nDNA (nuclear genome) to promote the blasts’ survival.

### 2.2. NFκB2 Regulates the Expression of Essential Nuclear Transcription Factors Responsible for Metabolic Plasticity in AML Blasts

Based on the above data on *NFκB2* and that the NFκB family is a group of prosurvival transcription factors that can rapidly respond to various stimuli and activate antiapoptotic genes [55], we hypothesized that the NFκB family, especially TKI-activated NFκB2, also mediates mitochondrial biogenic and metabolic pathways to support the higher energy demands of refractory blasts.

**Figure 2:**
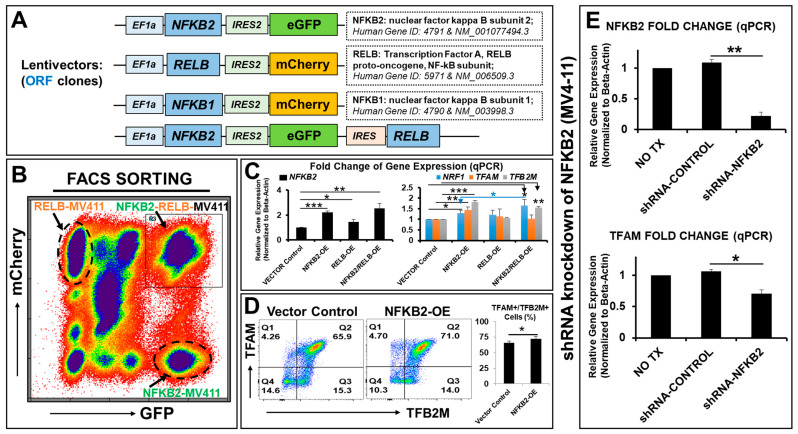
New transgenic cell lines of *NFκB*-family genes reveal that *NFκB2* overexpression (OE) promotes gene expressions of *TFAM, TFB2M* and *NRF1* in vitro. (N = 3). (**A**) Schematic diagram of lentiviral expression constructs containing open-reading frames (ORF) of human *NFκB2, RELB*, or *NFκB1* genes and *eGFP* or *mCherry* reporters, with promoters EF1a and IRES2, respectively; A bicistronic lenti-plasmid expresses both *NFκB2* and *RELB* controlled by EF1a and IRES promoters, respectively; Six new transgenic cell lines overexpressing *NFκB* family transgenes in leukemia blast MV4-11 were generated, including: *NFκB2*-*eGFP*-MV411, *NFκB1-mCherry*-MV411, *RELB*-*mCherry*-MV411, *NFκB2*-*RELB*-*eGFP*-MV411, *NFκB2*-eGFP/*RELB-mCherry*-MV411, *NFκB2*-eGFP/*NFKB1-mCherry*-MV411; and *GFP*-MV411 will be the vector control without transgene ORF insert; (**B**) All new cell lines were purified by FACS-sorting (see Materials and Methods); A representative FC plot shows multiple populations with eGFP and/or mCherry expression which were identified as *NFκB2*-*eGFP*-MV411, *RELB*-*mCherry*-MV411, *NFκB2-eGFP/RELB-mCherry*-MV411 before purification; (**C**) Gene expressions of *NFκB2, NRF1, TFAM* and *TFB2M* were analyzed by qPCR. Data of mRNA expressions show the fold change (normalized to *β-actin*) of genes; (**D**) Representative FC plots show protein expressions of TFAM and/or TFB2M in *NFκB2-eGFP*-MV411 and *GFP*-MV411 (vector control); Right table: Cumulative percentage data of viable TFAM+/TFB2M+ cells in different groups; (**E**) Gene expressions of *NFκB2* and *TFAM* after shRNA knockdown of NFκB2 in MV4-11 cells, analyzed by qPCR. Data of mRNA expressions show the fold change (normalized to *β-actin*) of *NFκB2* and *TFAM* in different treatment groups; Where applicable, data are means ± SEM. * *p* < 0.05, ** *p* < 0.01, *** *p* < 0.005.

To determine the physiologic role of NFκB2 in blast metabolism, we designed and utilized a lentiviral system to generate new transgenic leukemia cell lines overexpressing transgenes of *NFκB2* and its family members in MV4-11 cells (Figure 2A). Six new transgenic blast lines were created (see Supplementary Table S3 for detailed information), including *NFκB2-eGFP*-MV411, *NFκB1-mCherry*-MV411, *RELB-mCherry*-MV411, *NFκB2-RELB-eGFP*-MV411, *NFκB2-eGFP/RELB-mCherry*-MV411, and *NFκB2-eGFP/NFKB1-mCherry*-MV411, which had been further purified by cell sorting based on their fluorescent reporters of eGFP, mCherry, or overlapping eGFP and mCherry (Figure 2B). Another *GFP*-MV411 cell line was generated as the vector control without a transgene insert (ORF). The current study focused on the single or combinatory cell lines of the noncanonical NFκB pathway: NFκB2 (P100/P52) and RELB (the NFκB subunit, which associates with NFκB2 as RELB:P52 heterodimers in the nucleus) [46]. The overexpressed transgenes of *NFκB2* were experimentally validated using qPCR analyses (Left panel, Figure 2C).

Next, to understand how NFκB2 affects the blast metabolism and energy homeostasis, we examined these newly generated NFκB cell lines. We found that *NFκB2* overexpression significantly promoted the gene expressions of *TFAM, TFB2M*, and *NRF1* when compared to the lentivector control (right panel, Figure 2C). There was a significant additive effect in induction of the *NRF1* gene by *NFκB2-eGFP/RELB-mCherry*-MV411 when compared to *NFκB2-eGFP*-MV411 (right panel, Figure 2C). *NFκB2-RELB-eGFP*-MV411 (another cell line with bicistronic transgenes) confirmed the similar genetic effects observed in *NFκB2-eGFP/RELB-mCherry*-MV411. Furthermore, FC plot analyses confirmed the increased expressions of TFAM and TFB2M proteins in *NFκB2-eGFP*-MV411 when compared to the lentivector control (Figure 2D).

Furthermore, to determine whether NFκB2 is required for *TFAM* gene expression, we performed experiments of shRNA knockdown of *NFκB2* mRNA in MV4-11 cells. The qPCR analyses showed that there was significantly decreased gene expression of *NFκB2*, as was anticipated (an ~80% decrease versus scramble shRNA-control; Figure 2E). *TFAM* expression was also significantly decreased (an ~35% decrease versus scramble shRNA-control; Figure 2E). In summary, our data demonstrate that NFκB2 regulates the gene expressions of key transcriptional regulators of mitochondrial biogenesis and metabolic reprogramming in the blasts.

### 2.3. NFκB2 Was Localized in Human Mitochondria

**Figure 3:**
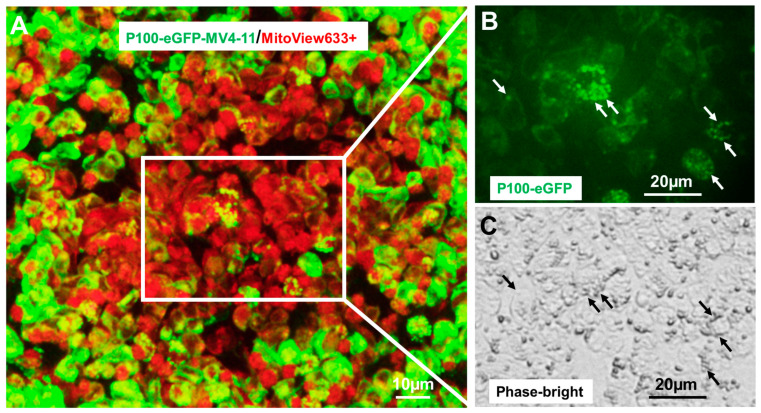
**Visualization of mitochondrial NFκB2 in MV4-11 blasts.** (**N = 3**)**.** (**A**) Generation of *P100-eGFP* (fusion)-MV4-11 cell line through the lentiviral system; Consistent with *P100-eGFP* (fusion)-HEK-293T cells, P100-eGFP was localized in cytoplasm of MV4-11. The immunocytochemistry (representative fluorescent images) was performed to show the co-localization of MitoView™633-stained mitochondria and GFP+ mitochondria at low magnification with a scale bar of 10 µm. (**B**,**C**) The white box of (**A**) was magnified to show many GFP+ mitochondria by a GFP-fluorescent image (indicated by white arrows, (**B**) and by a Phase-bright image of mitochondrial morphology (indicated by black arrows, (**C**) in these *P100-eGFP* (fusion)-MV4-11 cells with a scale bar of 20 µm.

If NFκB2 coordinates the metabolic adaptations in blasts, it is possible that NFκB2 can localize in mitochondria, like a small number of other nuclear TFs [56]. Different from traditional techniques (e.g., immunoelectron microscopy, co-immunoprecipitation, etc.) to identify nuclear TFs in mitochondria [57], we generated *NFκB2-eGFP* fusion reporter blast (MV4-11) cell lines to directly visualize the mitochondrial NFκB2 by utilizing genetic reporter constructs of *P100-eGFP*-fusion (full length *NFκB2* and NM_001077494.3 with eGFP fusion) and *P52-eGFP*-fusion (truncated *NFκB2*, NM_001077494.3, and 1-454aa with eGFP fusion). Consistent with the previous findings of NFκB2 (46), P100-eGFP was localized in the cytoplasm (Figure 3). Interestingly, many GFP+ mitochondria could be observed in P100-eGFP+ cells at low magnification (Figure 3A) and at higher magnification (Figure 3B,C). Also, we generated *P100-eGFP* (fusion)-HEK-293T cells (human embryonic kidney cells), which confirmed the existence of NFκB2 in other human mitochondria (Supplementary Figure S1A–C). Consistently, P100-eGFP was localized in the cytoplasm, while P52-eGFP was localized in the nuclei (Inset in Supplementary Figure S1A). Interestingly, many GFP+ mitochondria could be observed in P100-eGFP+ cells at low magnification (indicated by blue and white arrows in Supplementary Figure S1A) and at higher magnification (indicated by red arrows in Supplementary Figure S1B). The mitochondrial NFκB2 was also confirmed by immunocytochemistry showing the colocalization of MitoView633+ mitochondria and GFP+ P100 (indicated by white arrows in Supplementary Figure S1C) and the colocalization of DAPI-stained mitochondria and GFP+ P100.

To determine whether NFκB2 is imported into mitochondria or generated from mRNA by the organelle itself, we examined the changes of RNA transcripts in the mitochondria of TKI-treated blasts. We isolated their mitochondria (inset showing the magnified image in Supplementary Figure S1D) and analyzed their gene expressions using qPCR analyses (Supplementary Figure S1E). Consistent with the increased *NFκB2* mRNA from whole-cell samples, we found significantly increased *NFκB2* transcripts in isolated mitochondria from 80 nM GILT-treated MV4-11 cells (3.8-fold increase when compared to isolated mitochondria of NO TX control (Supplementary Figure S1E)). In contrast, there was no change in the *NFκB1* transcripts in isolated mitochondria between the NO TX and GILT-treated groups (Supplementary Figure S1E), while there was increased *NFκB1* mRNA in the whole-cell samples (Figure 1E).

### 2.4. NFκB2 Activated the Promoters of Blast Mitochondrial–Nuclear Dual Genomes

**Figure 4:**
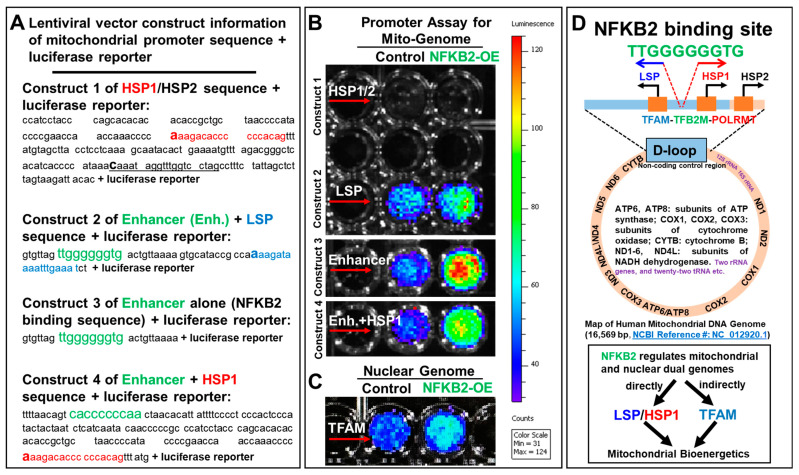
**NFκB2 activated promoters of dual mitochondrial-nuclear genomes in vitro.** (**N = 3**)**.** (**A**) Sequence information of lentiviral vector constructs (custom-built by GeneCopoeia) of mitochondrial LSP promoter (blue sequence), HSP1 promoter (red sequence) or HSP1/HSP2 promoter (underlined sequence) and a NFκB2-binding sequence (green sequence and named as Enhancer); (**B**,**C**) Promoter assays of mitochondrial genome (**B**) and nuclear genome (**C**) in blasts were performed (see details in the Materials and Methods); representative live images show luciferase activity in the supernatants of different experimental groups; (**D**) A schematic diagram illustrating the NFκB2-binding sequence on the D-loop of human mitochondrial DNA genome; Under pathologic or physiologic condition, NFκB2 may activate mitochondrial LSP promoter, HSP1/2 promoters and nuclear *TFAM* promoter to initiate mitochondrial biogenesis.

To understand whether NFκB2 plays a direct functional role in mitochondrial transcription, we performed transcriptional reporter assays. A changed expression of the construct (e.g., luciferase activity) following the context of NFκB2 stimulation or overexpression will reveal whether these regulatory mtDNA sequences can be activated by NFκB2 or not. Briefly, we lentivirally transduced LSP and HSP1/2 promoter (a putative regulatory sequence of mtDNA) reporters (with detailed construct and sequence information in Figure 4A) into *NFκB2-eGFP*-MV4-11 and *GFP*-MV4-11 cells (vector control) (detailed information of promoter cell lines can be found in Materials and Methods and Supplementary Table S4). Luciferase activity was measured in each cell line via the dual-luciferase assay kit (GeneCopoeia, Rockville, MD, USA) (Figure 4B). Increased luciferase radiance in the LSP-*NFκB2-eGFP*-MV4-11 cells when compared to LSP-*eGFP*-MV4-11 cells suggests a strong correlation between the ectopic gene expression of *NFκB2* and enhanced activities of the LSP promoter (lentivector construct 2 in Figure 4B), which is known to initiate the mitochondrial transcription [44].

To examine the exact binding site for NFκB2, we bioinformatically analyzed the D-loop sequence of the human mitochondrial genome and found the “TTGGGGGGTG” sequence (named as Enhancer and located at 440–431 of 16,569 bp nucleotides of the human mitochondrial complete genome (NC_012920.1)) between the LSP and HSP1 promoters, exactly matching the predicted noncanonical NFκB binding site of the nuclear genome [58]. The promoter assay of the “TTGGGGGGTG” sequence showing increased luciferase radiance in the Enhancer (alone)-*NFκB2-eGFP*-MV4-11 cells when compared to the Enhancer-*eGFP*-MV4-11 cells demonstrated that NFκB2 can bind this region and promote the luciferase activities (lentivector construct 3 in Figure 4B).

Because the “TTGGGGGGTG” sequence is located between the LSP and HSP1 promoters, we designed a new construct (lentivector construct 4 in Figure 4A) by adding the complementary sequence “CACCCCCCAA” of the Enhancer to the HSP1 promoter to determine whether NFκB2 can bind the reversed sequence and promote the luciferase activities of the HSP1 promoter. In contrast, our data show no luciferase activities of the HSP1/2 promoter (without the reversed sequence in lentivector construct 1 in Figure 4A), whereas adding “CACCCCCCAA” significantly promoted the luciferase activities of the Enhancer/HSP1 promoter in *NFκB2*-overexpressed MV4-11 cells when compared to the vector control (lentivector construct 4 in Figure 4B). Finally, consistent with the gain and loss of function tests (Figure 2), increased activities of the *TFAM* promoter (Figure 4C) demonstrated NFκB2′s regulatory role in the gene expression of *TFAM*, supporting our hypothesis that TKI-increased NFκB2 can regulate both mitochondrial and nuclear transcription systems directly (through promoting LSP- and HSP1-mediated transcription) and indirectly (through increasing TFAM) to undergo mitochondrial biogenesis and metabolic reprogramming to support AML relapse (Figure 4D). In summary, our data suggest that NFκB2 can directly initiate mitochondrial transcription.

### 2.5. The Triple Combination of Gilteritinib + SN52 + Oligomycin Effectively Eliminated AML Blasts by Inhibiting Their Mitochondrial Biogenesis and Functions

**Figure 5:**
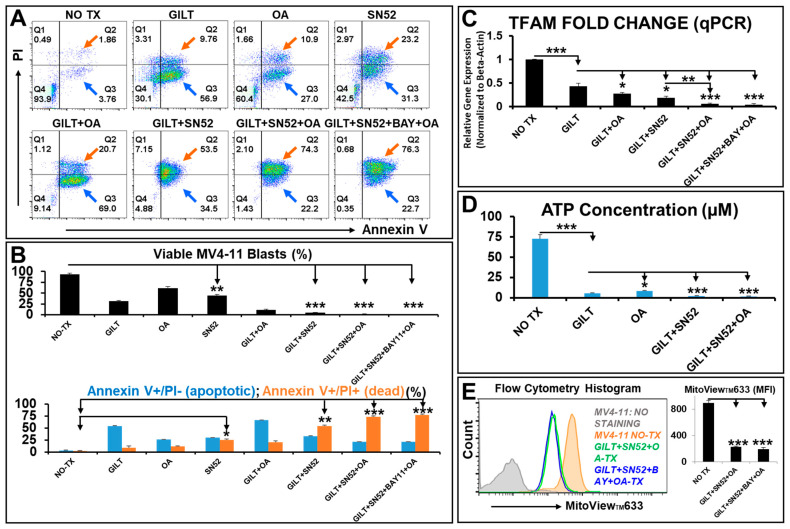
**Novel therapies eliminated AML blasts in vitro by inhibiting their mitochondrial biogenesis and functions.** (**N = 3**). (**A**) Representative FC plots of different experimental groups of MV4-11 cells with NO TX, 80 nM GILT, 100 nM oligomycin (OA), 15 µM SN52 (NFκB2-I), or 15 µM BAY11-7082 (NFκB-I), GILT + OA, GILT + SN52, GILT + SN52 + OA and GILT + SN52 + OA + BAY, which were analyzed by cell death biomarkers including Annexin-V (apoptosis), Propidium Iodide (PI, necrosis) and Annexin-V+/PI+ (dead); The blue arrows indicate Annexin-V+/PI- cells (early apoptotic); The orange arrows indicate Annexin-V+/PI+ cells (late-stage dying or dead cells); (**B**) **Upper panel:** Cumulative percentage data of viable MV4-11 blasts in different treatment groups (both Annexin-V negative /PI negative populations in the FC plots); **Lower panel:** Cumulative percentage data of Annexin-V+/PI- cells (blue column and indicated by blue arrows in plots of (**A**)), and Annexin-V+/PI+ cells (orange column and indicated by orange arrows in plots of (**A**)) in different treatment groups; (**C**) Gene expressions of *TFAM* were analyzed by qPCR. Data of mRNA expressions show the fold change (normalized to *β-actin*) of TFAM in different treatment groups; (**D**) ATP concentration was measured in MV411 cells of different treatment groups; (**E**) Representative FC histograms show expression of MitoView™633 in different treatment groups with NO TX, GILT + SN52 + OA and GILT + SN52 + BAY + OA; **Right table:** Cumulative MFI (mean fluorescence intensity) data of MitoView™633 expression in different treatment groups; Where applicable, data are means ± SEM. * *p* < 0.05, ** *p* < 0.01, *** *p* < 0.005.

Our data show that OA could sensitize blasts to GILT treatment with a ~10% increase in Annexin-V+/PI+ dead cells in the GILT + OA treatment (~20.7% Annexin-V+/PI+ cells) versus the GILT alone treatment (~9.76% Annexin-V+/PI+ cells) (Figure 5A). However, there remained sufficient viable blasts (~9% Annexin-V-/PI- cells) and early apoptotic MV4-11 (69% Annexin-V+/PI- cells) in the GILT + OA treatment (Figure 5A), which would be at high risk of relapsing [59]. Previously, we and other groups reported the NFκB2-mediated prosurvival pathways after AML treatments [9,48]. In this regard, we hypothesized that the simultaneous inhibition of NFκB2-mediated proinflammatory and prometabolic pathways could further improve the therapeutic efficacy of GILT + OA. Thus, we explored different in vitro combinations of GILT, OA, and NFκB inhibitors, including BAY11-7082 (BAY)—an inhibitor of the NFκB signaling pathway—and SN52—a potent NFκB2 inhibitor that can block RELB:P52 heterodimer formation and its nuclear import [60]. Also, ~15 µM SN52 was reported to be the most efficient concentration to induce cell death and enhance the radiosensitivity of cancer cells [60].

To evaluate the cytotoxic effect of the triplet therapies on blasts, we performed FC analyses of the cell death biomarkers, including Annexin-V (apoptosis), Propidium Iodide (PI, necrosis), and Annexin-V+/PI+ (late-stage dying or dead) (MEBCYTO Apoptosis Kit: MBL 4700). FC results strongly supported our hypothesis by showing that the administration of SN52 alone (23.2%) could significantly promote more Annexin-V+/PI+ dead cells than GILT (9.76%) or OA alone (10.9%) (Figure 5A,B). The supplementation of SN52 to GILT treatment was found to further promote the blasts to yield a much higher percentage of Annexin-V+/PI+ dead cells (53.5%) when compared to GILT + OA (20.7%) (Figure 5A,B). Additionally, we found that the combination of GILT + SN52 + OA or GILT + SN52 + BAY + OA promoted the blasts to yield the highest percentage of Annexin-V+/PI+ dead cells (74.3% and 76.3%, respectively; Figure 5A,B). However, the addition of BAY11-7082 (an NFκB1 inhibitor) to the GILT + SN52 + OA treatment had only a marginal additive effect on the PI expression (FC plots in Figure 5A and lower table in Figure 5B). While all treatments reduced the viable MV4-11 blasts, the GILT+ SN52 (4.88% Annexin-V-/PI- viable cells), GILT + SN52 + OA (1.43% Annexin-V-/PI- viable cells), and GILT + SN52 + BAY + OA (0.35% Annexin-V-/PI- viable cells) treatments had significantly lowered viable MV4-11 blasts when compared to other treatments such as GILT alone (30.1% Annexin-V-/PI- viable cells; upper table of Figure 5B).

To determine the mechanisms underlying the therapeutic effects of the triple combination, we performed a qPCR to analyze the gene expression of *TFAM*. All combination therapies significantly reduced *TFAM* when compared to the NO TX control, including GILT alone (57% decrease versus NO TX), GILT + OA (73% decrease versus NO TX), GILT + SN52 (81% decrease versus NO TX), GILT + SN52 + OA (99.94% decrease versus NO TX), and GILT + SN52 + BAY + OA (99.96% decrease versus NO TX) (Figure 5C). Moreover, the SN52-based combination/triplet therapies had stronger inhibitory effects on *TFAM* when compared to GILT alone (Figure 5C).

To examine whether the triplet therapy can effectively inhibit the energy conversion of the blasts, we performed a functional test by measuring the ATP concentration in treated blasts (Figure 5D). Consistent with the significant reduction in *TFAM* expression, GILT + SN52 (97.1% decrease versus NO TX) and GILT + SN52 + OA (97.7% decrease versus NO TX) had stronger inhibitory effects on ATP production when compared to GILT alone (92.3% decrease versus NO TX) and OA alone (88.7% decrease versus NO TX) (Figure 5D). Furthermore, FC analyses of MitoView™633 expression confirmed that the combination of GILT + SN52 + OA and GILT + SN52 + BAY + OA had significantly lower MitoView™633 expression when compared to NO TX, suggesting severe damage to the mitochondrial membrane potential (MMP) in treated MV4-11 cells (Figure 5E).

### 2.6. The Triplet Therapy Exhibited a Robust Antileukemia Effect against Primary AML Blasts with Minimum Toxicity to the Control Cells of 10 Patients Ex Vivo

**Figure 6:**
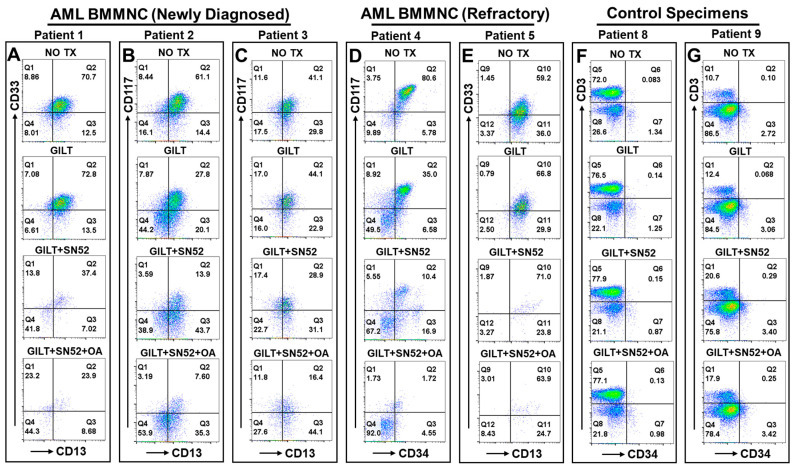
The triplet therapy displayed a potent anti-leukemia effect on *FLT3*-mut blasts of newly diagnosed and refractory AML patients ex vivo. (N = 10). (**A**–**C**) Representative FC plots of viable blasts (gated on viability dye negative populations) from different experimental groups of bone marrow mononuclear cells (BMMNC) from three newly diagnosed AML patients with NO TX, 80 nM GILT, 80 nM GILT + 15 µM SN52, and 80 nM GILT + 15 µM SN52 + 100 nM OA, which were analyzed by CD13, CD33 and CD117 (c-kit) according to patients’ clinical profiles; (**D**,**E**) Representative FC plots of viable blasts (gated on viability dye negative populations) from different experimental groups of BMMNC specimens from two refractory AML patients with NO TX, 80 nM GILT, 80 nM GILT + 15 µM SN52, and 80 nM GILT + 15 µM SN52 + 100 nM OA, which were analyzed by CD117, CD34, CD33 and CD13 according to patients’ clinical profiles; (**F**,**G**) Representative FC plots of viable cells (gated on viability dye negative populations) from different experimental groups of peripheral blood (PB) specimens from two healthy patients with NO TX, 80 nM GILT, 80 nM GILT + 15 µM SN52, and 80 nM GILT + 15 µM SN52 + 100 nM OA, which were analyzed by CD3 and CD34.

We performed a preliminary ex vivo experiment to evaluate the therapeutic effect of the triple combination on primary AML specimens (clinical profiles of patients available in Table 1), including bone marrow (BM) mononuclear cells (BMMNCs) from *FLT3*-mut AML patients (newly diagnosed shown in Figure 6A–C; Patient 6 in Supplementary Figure S2A; refractory shown in Figure 6D,E; Patient 7 in Supplementary Figure S2B). Our FC results of patients 1–3 (newly diagnosed) showed that the supplementation of SN52 in GILT treatment could more effectively reduce the viable CD33 + CD13+ or CD117 + CD13+ blasts (37.4%, 13.9%, and 28.9%) when compared to GILT alone (72.8%, 27.8%, and 44.1%) (Figure 6A–C). Furthermore, the triple combination of GILT + SN52 + OA significantly reduced the blasts (23.9%, 7.6%, and 16.4%) when compared to NO TX (70.7%, 61.1%, and 41.1%) and even more than the GILT + SN52 combination mentioned above (Figure 6A–C) (right table in Supplementary Figure S2A).

Next, we examined the therapeutic efficacy of the triplet therapy on refractory patient samples. AML patient 4 (Figure 6D) was a 24-year-old male *FLT3*-mut AML patient who had received midostaurin treatment and was admitted for worsening symptoms with a >80% blast population expressing both CD34 and CD117 (c-kit). Our FC results showed that the supplementation of SN52 in GILT treatment could effectively reduce the viable CD117 + CD34+ blasts (10.4%) when compared to GILT alone (35%) (Figure 6D). Furthermore, the triple combination of GILT + SN52 + OA eliminated the blasts (1.72%) when compared to NO TX (80.6%) and more than just the GILT + SN52 combination (10.4%) (Figure 6D). In another refractory Patient 5, our FC results confirmed that the triplet therapy eliminated a large quantitative population of CD33 + CD13+ blasts despite similar CD33 + CD13+ percentages compared to the other three groups (Figure 6E).

However, given that the mitochondrial OXPHOS system is essential for ATP/metabolite production to support the physiologic function of all cells, the toxic side effects of mitochondria-targeted therapies present a major obstacle for finding effective dosing to treat cancers and a potential roadblock for this therapy to work safely in patients [61,62]. In this regard, we examined the cytotoxcity of the triplet therapy at the current dose on healthy hematopoietic cells ex vivo. Our FC results of three healthy patients’ PB cells showed that both CD3+ T cells and CD34+ hematopoietic progenitor cells displayed similar cellular patterns between the NO TX and combination or the triplet therapies, with no apparent reduction in healthy hematopoietic cells (Figure 6F,G and Supplementary Figure S2C). In summary, our data show that the triplet of GILT + SN52 + OA performed robust antileukemia effects in vitro and ex vivo through disrupting mitochondrial biogenesis, energy conversion, and the MMP of AML blasts.

## 3. Discussion

Recently, we found a group of transient CD44 + pBAD+ blasts that undergo intrinsic homeostatic adaptation to elude treatment and initiate disease relapse [8,9]. To continue our effort to develop effective therapies to overcome this novel drug resistance, we investigated the mechanism underlying the metabolic plasticity of refractory blasts. Here, our data demonstrate that targeted therapies damaged blast mitochondria, leading to mitochondrial–nuclear genomic reprograming in the refractory blasts. Mechanistically, NFκB2 was found to coordinate the transcriptional adaptation of the dual genomes responsible for mitochondrial biogenesis and metabolic plasticity in AML blasts. Based on our mechanistic discovery, we explored a novel triplet therapy that effectively eliminated AML blasts by abolishing *TFAM* and ATP production with minimum toxicity to healthy cells in vitro.

### 3.1. Noncanonical NFκB2 Is a Single Transcription Factor Individually Regulating the Dual Mitochondrial–Nuclear Genomes Essential for Mitochondrial Biogenesis and Metabolic Reprogramming in AML

**Figure 7:**
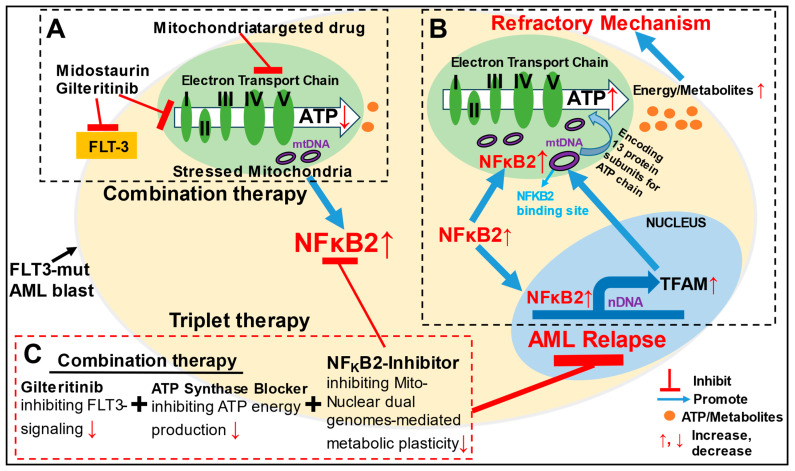
**Overview of inhibiting NFκB2-mediated mitochondrial-nuclear transcriptional adaptation to overcome AML relapse.** (**A**) Mitochondria-targeted drugs such as oligomycin, an ATP synthase blocker (Complex V) can improve the therapeutic efficacy of TKIs; however, mitochondrial damage and stress result in the activation of pro-survival transcription factors of noncanonical NFκB2 in refractory blasts. (**B**) Notably, increased NFκB2 can promote the nuclear gene (nDNA) expression of *TFAM*, *TFB2M*, and *NRF1*, which are known to be essential for mitochondrial biogenesis and metabolic plasticity, in addition to its activation of a group of pro-inflammatory and pro-survival factors such as CD44/cytokines/receptors (as we reported previously), thus leading to a large cascading effect on homeostatic adaptation to support blast survival and AML relapse energetically and metabolically. Increased NFKB2 enters the mitochondria and binds specific “TTGGGGGGTG” region of D-loop to promote biogenesis and biosynthesis to support the respiratory chain for ATP/metabolites production. (**C**) In this regard, we developed a novel triplet strategy blocking FLT3 signaling, targeting blast mitochondrial energy conversion, and inhibiting NFκB2 simultaneously, which can effectively promote the terminal death of AML blasts and prevent AML relapse.

The mammalian mitochondrial proteome is under dual genomic control, with 99% of its proteins encoded by the nDNA and 13 proteins originating from the mtDNA [31]. To fulfill the metabolic demand of cancer cells, the mitochondria must perform functions at multiple levels, including altering the bioenergetic production of ATP and biosynthesizing fundamental molecules required for tumorigenic growth and metastasis [14]. For such a significant role, the mitochondria require full cooperation from the nDNA through the continuous anterograde or retrograde communication of signals and the transportation of materials [33]. However, detailed molecular and genetic mechanisms of the crosstalk between dual genomes are not yet completely understood during physiologic and pathologic states [34]. Previously, the PPARγ coactivator-1 (PGC-1) family, a group of transcriptional coactivators, has been found to be differentially expressed in response to different extracellular signals [63]; however, they depend on other transcription effectors such as NRF-1 to modulate the metabolic adaptations and activate TFAM and TFB2M to govern mitochondrial gene expressions [53]. Thus, it is of interest to determine whether there is a single signal-responding transcription factor (rapidly activated) that can coordinate the metabolic reprogramming of both mitochondrial and nuclear transcription systems under physiologic and pathologic conditions. The identification and characterization of such a transcriptional regulator and its interaction networks is critical for the development of effective therapies to specifically cut off the circuitry between dual mitochondrial–nuclear genomes (crosstalk shown in Figure 7B) to overcome treatment resistance and improve AML prognosis [15,64].

Like the mitochondrion, the NFκB family is a group of ancient molecules in our biological phylogenetic tree, contributing substantially to cellular functions and cell survival [65]. There has thus been literature speculating about the localization of NFκB members in the mitochondrion [66]. RELA (P65), a canonical NFκB subunit, was reported to downregulate energy homeostasis [67] in the mitochondria but also upregulate mitochondrial respiration [68]. However, due to the limitations of traditional techniques [57] and the existence of nuclear mtDNA segments [69], the results have been inconclusive without many follow-up studies since then to confirm the observation and uncover the specific binding site sequence of mtDNA. In this study, we utilized genetic approaches to provide definitive evidence of the mitochondria-localizing NFκB2 protein and define its targeted sequences of mtDNA (Figure 3 and Figure 4). First, our newly generated P100-eGFP fusion reporter cell line directly visualized the NFκB2 proteins in the mitochondria of MV4-11 blasts (Figure 3A–C and Supplementary Figure S1). Currently, only a small number of transcription factors have been reported to recognize the protein-coding region outside the regulatory domain (D-loop) [25]. Our promoter binding assays demonstrated that NFκB2 directly binds to a specific “TTGGGGGGTG” region between the LSP and HSP1 promoters of the mitochondrial D-loop and enhanced their promoter activities in vitro (Figure 4). Furthermore, NFκB2 was found to activate the promoter of nuclear *TFAM* in vitro (Figure 4C), which is consistent with the gain/loss of function tests (Figure 2) and reveals the requirement of NFκB2 for *TFAM* expression. Based on current knowledge, human mitochondrial transcription depends entirely on a nuclear DNA-encoded transcription machinery involving TFAM that binds LSP and HSP promoters, unwinds mtDNA, and recruits the TFB2M and POLRMT (a mitochondrial RNA polymerase) to initiate mtDNA transcription [44] (Figure 4D). Thus, it is important to investigate whether NFκB2 activates LSP/HSP/TFAM promoters in vivo, as well as how NFκB2 interacts with the TFAM-TFB2M-POLTMT machinery to initiate and maintain the mtDNA transcription. Altogether, we provide the first results of evidence showing that NFκB2 is a master regulator of the dual mitochondrial–nuclear transcription systems required for blast metabolism.

In regard to how NFκB2 enters the mitochondrion, we speculate that NFκB2 accesses the mitochondria through mitochondrial permeability transition pores (mPTP—a multiprotein channel complex) under pathologic conditions [70,71] (Figure 7B). Interestingly, we also found upregulated NFκB2 transcripts in the isolated mitochondria of GILT-treated MV4-11 cells (Supplementary Figure S1E). It is possible that these NFKB2 transcripts localize to the mitochondrial surface to facilitate the efficient translocation of nascent peptides (synthesized in the cytoplasm) into the organelle [72]. Thus, to prevent NFκB2-initiated mitochondrial biogenesis and subsequent events, we reasoned that it would be important to identify the entrance route for either the NFκB2 protein or mRNA into mitochondria and determine the mitochondrial translation machinery specific to *NFκB2* transcripts [73]. In summary, our evidence suggests that NFκB2 and its binding region “TTGGGGGGTG” can be therapeutic targets to treat AML (Figure 7B).

### 3.2. Inhibition of the Key Transcriptional Regulator of Dual Genomes Responsible for Metabolic Plasticity Is a Fundamental Strategy toward Eliminating AML Blasts

Previously, we exploited the Warburg effect, where cancer cells preferentially uptake glucose to produce lactate for energy despite an oxygen abundance [16] to develop a less-toxic vitamin D-based strategy of bottlenecking the glycolytic pathways of leukemia cells [74]. However, treatment-resistant blasts hijacked mitochondria and their quality control system to replenish key metabolites and maintain metabolic homeostasis [51], suggesting the importance of therapeutically targeting the dual genomic reprogramming required for the metabolic plasticity of cancer cells, which is an advance that will address (potentially avoiding long delays in understanding) the compensatory relationship between glycolytic metabolism in the cytosol and OXPHOS system in the mitochondria [75].

Currently, there are ongoing mono and combination therapies targeting mitochondrial respiratory complexes or metabolites at the stage of experimental evaluation and clinical trials for AML, which include inhibitors of BCL-2, enzymes in the glycolysis and citric acid cycle, the electron transport chain (e.g., metformin), and glutamine or fatty acid pathways [45]. However, based on our mechanistic findings, we hypothesize that these metabolism-targeted strategies would eventually be compromised by adaptive therapy tolerance [76] through the activation of mitochondrial–nuclear compensatory reprogramming (such as the upregulation of nuclear *NRF1* shown in Figure 1C,D and NFκB2-mediated mitochondrial biogenesis shown in Figure 4D) to bypass such transient suppression and jeopardize therapeutic efficacy (Figure 7A). This potentially leads to the failure of clinical trials involving multiple mitochondrial metabolism drugs in addition to their clinical toxicity [77].

To experimentally validate the refractory mechanism caused by oligomycin treatment, we investigated other mitochondria-targeted drugs, including metformin (inhibitor of ETC complex I) and bedaquiline (another inhibitor of ATP synthase: ETC complex V). These repurposed FDA-approved drugs displayed similar antileukemia effects on MV4-11 cells; however, like oligomycin, metformin and bedaquiline also significantly increased NFKB2 and activated NFKB2-mediated prosurvival pathways in the refractory blasts. In this regard, we reasoned that targeting the key transcriptional regulators, such as NFκB2, and shutting down NFκB2-mediated mitochondrial–nuclear genomic crosstalk responsible for metabolic homeostasis could be more effective in inhibiting blast metabolism and overcoming drug resistance when treating AML than targeting the individual components of mitochondrial or glycolytic pathways alone [78,79]. To test this hypothesis, we explored a proof-of-concept triplet strategy (Figure 7C), including targeting intracellular signaling pathways (TKIs), blocking mitochondrial ATP synthase/OXPHOS (OA), and disrupting NFκB2-mediated metabolic reprogramming (SN52) in vitro. The triplet therapy of GILT + SN52 + OA eliminated all blasts in vitro by promoting most blasts into late-stage apoptosis and necrosis (both Annexin-V+/PI+). These actions greatly reduce the relapse risk of numerous blasts at the early stage of apoptosis (Annexin-V+/PI-, Figure 5B) [59] by effectively shutting down *TFAM* and ATP production (Figure 5C–E). Furthermore, the triplet therapy exhibited a robust antileukemia effect against the primary blasts of both newly diagnosed and refractory AML patients ex vivo (Figure 6A–E and Supplementary Figure S2A,B), suggesting the importance of shutting down dual mitochondrial–nuclear genomes essential for encoding many protein subunits for the OXPHOS system and metabolic reprogramming [31]. Finally, mtDNA replication stress and injured mitochondria have recently emerged as key drivers of inflammatory responses associated with pathogenic states or cell death by releasing immunostimulatory mtDNA-TFAM fragments, which could be a therapeutic target to improve cancer treatment [80,81]. Based on our previous findings, the triplet therapy can potentially suppress such mitochondrial-driven prosurvival inflammation [9].

The current study has potential ramifications for targeting cancer metabolism; however, it has several limitations that should be addressed in the future. Accordingly, additional proteomics and metabolomics should be employed in future functional studies to validate our transcriptional characterization of the metabolic plasticity in TKI-resistant blasts. NFκB transcription factors and the signaling pathways that activate them are one of the most important central regulators in inflammation, which play a critical role in cancer development and progression and are therefore an excellent target for cancer therapy [82]. Among many agents developed to target NFκB pathways, very few drugs have received approval for administration in AML patients so far [83], suggesting that there are complexities and challenges in clinical drug development rather than the proof-of-concept exploration of a NFκB2 inhibitor in the present study. Thus, the treatment efficacy (e.g., long-term effect), dose, and off-target toxicity of this unconventional triplet therapy, which is designed to target the principle of cancer metabolism, need to be evaluated and optimized in the next stage of investigation using primary patient blasts-derived AML murine models in vivo [84].

Throughout the lengthy process of human evolution, mitochondria have metamorphosized their status from intracellular parasites to essential regulators of metabolism, stress responses, and cell death [85,86], which involve a relatively small number of nuclear TFs (e.g., P53, cAMP Response Element-Binding Protein (CREB), and STAT3) that constantly monitor the cellular conditions to finetune their metabolic activities and coordinate adaptive responses for life or death [56]. Now, NFκB2 and its transcript join this exclusive list, with definitive evidence of individually regulating dual genomes; however, their roles and interactomes in mitochondrial biology (physiologic processes) and heterogeneous mitochondrial diseases (pathogenesis) remain to be uncovered.

In summary, we provide evidence for the first time that noncanonical NFκB2 is a single transcriptional regulator of the dual genomes responsible for mitochondrial biogenesis and metabolic plasticity in leukemia cells. The proof of principle of the triplet therapy ex vivo indicates the success of targeting NFκB2-mediated multiple proleukemia pathways to overcome AML relapse. Although many challenges remain for cancer medicine, promising outcomes to improve AML prognosis are emerging.

## 4. Materials and Methods

The list of reagents and transgenic cell lines, including manufacturers and catalogs of antibodies, kits, primers, and lentiviral plasmids, are available in the Supplementary Tables S1–S4. Detailed information of plasmids can be obtained from the corresponding author. Replicates (N = 3) were performed for all experiments.

### 4.1. Primary Patient Specimens

*FLT3*-mut AML bone marrow (BM) mononuclear cells (BMMNC) and healthy peripheral blood (PB) specimens (Patients 1–10, Table 1) were obtained from the Biospecimen Banks of Loma Linda University Medical Center (LLUMC) and City of Hope National Medical Center (COHNMC). All donor patients signed an informed consent form. Sample acquisition was approved by the Institutional Review Boards at the LLUMC and the COHNMC in accordance with an assurance filed with and approved by the Department of Health and Human Services, and it (IRB#:58238, 10-11-2023) met all requirements of the Declaration of Helsinki.

### 4.2. Cell Culture

MV4-11 (ATCC CRL-9591) is a human-derived AML blast cell line with *FLT3* mutation. The MV4-11- and MV4-11-based transgenic cell lines were cultured in RPMI-1640 medium (Hyclone, Thermo Scientific, Waltham, MA, USA), and they were supplemented with 10% heat-inactivated fetal bovine serum (FBS, HyClone) and 1% penicillin/streptomycin. Cells were grown at 37 °C in a humidified atmosphere containing 5% CO_2_.

### 4.3. In Vitro and Ex Vivo Treatment of AML Blasts

The list of inhibitors (I), abbreviations, manufacturers, and catalog numbers (#s) can be found in Supplementary Table S1. Gilteritinib (GILT) is a second-generation FLT3 inhibitor. As a single agent to treat blasts in vitro, a single dose of 80 nM of GILT was added to 1 mL of 1 × 10^6^ MV4-11 cells in 24-well plates based on our previous report [8]. As combination agents to treat blasts in vitro, one dose of 80 nM GILT with one dose of either 100 nM oligomycin (OA), 15 µM SN52 (NFκB2-I, an optimized dose based on the previous report [60]), or 15 µM BAY11-7082 (NFκB-I) was added to 1 mL of 1 × 10^6^ MV4-11 cells, 1 × 10^6^ healthy PB cells, or 2–5 × 10^5^ primary AML blasts for each experimental group in 24-well plates. Two days after the one-dose treatment, cells were then collected for further analyses.

### 4.4. Generation of New Transgenic Cell Lines In Vitro (See Details in Supplementary Table S3)

(a) *Preparation of lentiviral particles in vitro*

The detailed information of lentiviral transfer plasmids (including custom-built constructs) can be found in Supplementary Table S3. Lentiviruses were prepared as previously described [51]. Briefly, HEK-293T cells were cultured in complete Dulbecco’s Modified Eagle Medium (DMEM, Gibco, Waltham, MA, USA) containing 10% FBS and 100 U/mL penicillin/streptomycin. When the cells were 70–80% confluent, the culture media was replenished, and a transfection solution containing envelope, packaging, and transfer plasmids (e.g., *NFκB2* or *GFP*, etc.) was added dropwise to the cells. Afterwards, the cells were cultured at 37 °C and 5% CO_2_ for 48 h, filtered through a 0.45 µm filter, and centrifuged at 4800× *g* at 4 °C for 24 h. The virus pellet was reconstituted in PBS containing 5% glycerol and titrated.

(b) *NFκB family gene-overexpressed MV4-11 cell lines*

The lentiviral transfer plasmids (Supplementary Table S3) contain a full-length open reading frame (ORF) of human *NFκB2* (GeneCopoeia catalog#: EX-Z4293-Lv225, Rockville, MD, USA), *NFκB1* (GeneCopoeia catalog#: EX-F0208-Lv224), *RELB* (GeneCopoeia catalog#: EX-G0029-Lv224), and *NFκB2-RELB* bicistronic plasmid (custom-built by GeneCopoeia with catalog#: CS-Z4293-Lv225-01). The ORFs of *NFκB2* (NM_001077494.3), *NFκB1* (NM_003998.3), *RELB* (NM_006509.3), and *eGFP* or *mCherry* reporters are controlled by the EF1a and IRES2 promoters, respectively. A *GFP* empty vector (GeneCopoeia catalog#: EX-NEG-Lv225) was used as the vector control. The MV4-11 cells were transduced with the lentivirus at a multiplicity of infection (MOI) of 5. Twenty-four hours later, the virus was removed, and the culture medium was replenished. The new cell lines were further experimentally validated via fluorescence microscopy, gene expression, flow cytometry, and purified by fluorescence-activated cell sorting (FACS; Research Core Facility, School of Medicine, University of California, Riverside).

(c) *NFκB2 (P100)-eGFP and (P52)-eGFP reporter cell lines*

The lentiviral transfer plasmids (Supplementary Table S3) containing *NFκB2* (NM_001077494.3) or truncated sequence (NM_001077494.3, 1-454 amino acids (aa)) fused with *eGFP* were generated as *P100-eGFP* and *P52-eGFP* reporters and controlled by the EF1a promoter (custom-built by GeneCopoeia, Rockville, MD, USA with catalog#: CS-Z4293-Lv224-01 and CS-Z4293-Lv224-02). HEK-293T cells were transfected by these plasmids to generate *P100-eGFP* and *P52-eGFP* reporter cell lines (*P100-eGFP* (fusion)-HEK-293T and *P52-eGFP* (fusion)-HEK-293T, Supplementary Figure S3), which were further validated using microscopic and qPCR analyses. Also, we generated a *P100-eGFP* (fusion)-MV4-11 cell line using lentiviral transduction of a *P100-eGFP* reporter (custom-built by GeneCopoeia with catalog#: CS-Z4293-Lv224-03: without mCherry, Figure 3A), which confirmed the phenomenon of mitochondrial NFκB2 in the *P100-eGFP* (fusion)-HEK-293T.

(d) *NFκB2-knockdown MV4-11 cell lines*

A short hairpin RNA (shRNA) was used to knockdown *NFκB2* (*p100/52*) in MV4-11 cells (Supplementary Table S3). MV4-11 cells were grown in 6-well plates and transduced with human shRNA-*NFκB2* lentiviral particles, which contain 4 sets of unique 29mer target-specific shRNA targeting *NFκB2* (*P100/P52*) (Catalog#: TL311187V, OriGene, Rockville, MD, USA). MV4-11 cells transduced with scrambled shRNA lentiviral particles were used as control (shRNA-Control). Positively transduced cells were selected using puromycin (2 μg/mL). The qPCR experiments were performed to confirm *NFκB2* knockdown and examine genetic changes.

### 4.5. Promoter Assays of Mitochondrial Genome (LSP and HSP) and Nuclear Genome (TFAM) In Vitro

The detailed information of promoter sequence and cell lines can be found in Figure 4A and Supplementary Table S4. Briefly, lentiviral plasmids containing the promoter clone with Gaussia luciferase reporter for human HSP1/2 (194 base pair (bp) nucleotides; named as lentivector construct 1), LSP (60 bp nucleotides; named as lentivector construct 2), NFκB2 binding site (named as Enhancer, 27 bp nucleotides, and lentivector construct 3), and Enhancer/HSP1 (163 bp nucleotides; named as lentivector construct 4) for mitochondrial genome were custom built and acquired from GeneCopoeia (Rockville, MD, USA). The DNA sequences of human LSP and HSP1/2 promoters (Figure 4A) were based on a previous report [32]. The sequence of NFκB2 binding site (Enhancer) in mitochondrial genome was bioinformatically analyzed and examined based on the information of noncanonic NFκB binding on the nuclear genome [58]. A lentiviral plasmid containing the promoter clone with Gaussia luciferase reporter for human *TFAM* (NM_001270782) was acquired from GeneCopoeia (Catalog#: HPRM61407-LvPG04). The preparation of the lentivirus of the promoter clones was performed by using the same protocol for the lentivirus for *NFκB* family genes. The HSP1/2, LSP, Enhancer, Enhancer/HSP1, and *TFAM* promoter reporters were lentivirally transduced into *GFP* + MV4-11 (vector control) and *NFκB2-eGFP*-MV4-11 cell lines to generate HSP1/2-*GFP*-MV4-11 and HSP1/2-*NFκB2-eGFP*-MV4-11 blasts, LSP-*GFP*-MV4-11 and LSP-*NFκB2-eGFP*-MV4-11 blasts, Enhancer-*GFP*-MV4-11 and Enhancer-*NFκB2-eGFP*-MV4-11 blasts, HSP1(+Enhancer)-*GFP*-MV4-11 and HSP1 (+Enhancer)-*NFκB2-eGFP*-MV4-11 blasts, and *TFAM-GFP*-MV4-11 and *TFAM-NFκB2-eGFP*-MV4-11 cells in vitro. The detection of promoter activities in the supernatants of these transgenic cell lines (equal numbers of cells in experimental groups) was performed using the Secrete-Pair™ Dual Luminescence Assay Kit (GeneCopoeia, Rockville, MD, USA). Images were acquired through a high-resolution CCD camera (Perkin Elmer IVIS Lumina III, Waltham, MA, USA).

### 4.6. Flow Cytometry (FC)

Cells were harvested and examined for the expression of viability dye, cell surface biomarkers, and intracellular proteins using multichromatic FC, as previously described [87]. MitoView™633 is a fluorescent mitochondrial dye that is dependent on mitochondrial membrane potentials (MMP, ΔΨm), which are essential for transport of ions, protons, and the respiratory chain of ATP production [88], and is designed for staining mitochondria in live cells (Biotium, Fremont, CA, USA, with Catalog#: 70055). As a result, lower MitoView™633 expressions (Mitoview™633- (higher percentage negative or weak) or Mitoview™633+ (lower percentage positive)) could indicate damaged MMP and, consequently, a reduction in oxidative respiratory and transport capabilities of the mitochondria, providing one method for evaluating the therapeutic effect on AML blasts. The viability dye used in this study was Fixable Viability Dye eFluor™ 780 (eBioscience, San Diego, CA, USA, with Catalog#: 65-0865-14). Briefly, for FC staining, after the staining of viability dye, about 1 × 10^4^ ~ 10^6^ cells in 100 µL FC buffer (PBS containing 1% FBS and 0.05% sodium azide) were stained with various fluorescence-conjugated antibodies specific for the desired cell surface proteins at 4 °C for 30 min. Annexin V apoptosis detection kit was used for identifying apoptotic and necrotic cells (MBL, Schaumburg, IL, USA, with Catalog#: 4700). Concentrations of antibodies and dyes were applied according to the manufacturers’ recommendations. Finally, the stained cells were detected on the BD FACSAria II. FC data were analyzed using the FlowJo software (v10.6, Tree Star Inc., Ashland, OR, USA).

### 4.7. Mitochondrial Isolation

MV4-11 cells treated with or without TKIs were collected. Mitochondria were isolated by using the previously reported protocol [89]. Isolated mitochondria were experimentally validated using microscopic and MitoTracker analyses.

### 4.8. RNA Isolation and Real-Time Polymerase Chain Reaction (qPCR) Analysis

RNA isolation and qPCR analysis of gene expressions were performed as previously described [90]. Total RNA of cells or isolated mitochondria were isolated using the RNeasy Micro Kit (Qiagen, Germantown, MD, USA) according to the manufacturer’s instructions. First-strand cDNA was synthesized using the SuperScript III Reverse Transcriptase (Invitrogen; Life Technologies, Waltham, MA, USA). With an Applied Biosystems 7900HT Real-Time PCR machine (Waltham, MA, USA), qPCR was performed and analyzed. Primers used in this study are available in Supplementary Table S2. The PCR conditions were 10 min at 95 °C followed by 40 cycles of 10 s at 95 °C and 30 s at 60 °C. The relative expression level of a gene was determined using the ΔΔCt method and normalized to β-actin.

### 4.9. ATP Assay

We collected treated MV4-11 cells and measured their ATP levels by using an ATP assay colorimetric kit (Abcam, Waltham, MA, USA). The procedures were done following the manufacturer’s instructions, which are based on phosphorylation of glycerol and can be colorimetrically quantified (OD = 570 nm).

### 4.10. Imaging Acquisition

Immunocytochemistry and imaging acquisition were performed as previously described [74]. Briefly, concentrations of antibodies and dyes were applied according to the manufacturers’ recommendations. Phase-bright and fluorescent images were taken using an Olympus 1X71 inverted microscope and were processed using Olympus cellSens Dimension 1.15 Imaging Software (v2.2) (Tokyo, Japan).

### 4.11. Statistical Analysis

Statistical analyses were performed with GraphPad Prism software (Prism 5.0, San Diego, CA, USA). The quantitative analyses were analyzed using a one-tailed or two-tailed unpaired *t* test for comparison of two groups, or one-way or two-way ANOVA test for comparison of three or more groups. All values are presented as mean ± SEM. Results were considered statistically significant when the *p* value was <0.05.

## Figures and Tables

**Table 1:** List of AML patients and Healthy patients.

Sample ID	Diagnosis	Sex	Age	Gene Mutation
AML Patient 1	AML (Newly diagnosed)	M	76	*FLT3* (c.2503G > T; p.D835Y): Allele Frequency 41%
AML Patient 2	AML (Newly diagnosed)	M	62	*FLT3*-ITD: Level = 0.98
AML Patient 3	AML (Newly diagnosed)	M	75	*FLT3*-ITD: Allele Frequency 0.27%
AML Patient 4	AML (Refractory)	M	24	*FLT3*-ITD; 46,XY,t(6,11)(q27;q23) [11]/46,XY [9]
AML Patient 5	AML (Refractory)	M	69	*FLT3*-ITD: Signal Ratio 0.89 (c.1789delins25; p.Y597delins9) (23% allele)
AML Patient 6	AML (Newly diagnosed)	M	52	*FLT3*-ITD: *FLT3* Internal Tandem Duplication (ITD) confirmed by PCR with Signal Ratio 0.12.
AML Patient 7	AML (Refractory)	F	65	*FLT 3*-ITD: Signal Ratio 0.57
Healthy Patient 8	N/A	M	50	Normal
Healthy Patient 9	N/A	M	55	Normal
Healthy Patient 10	N/A	F	40	Normal

## Data Availability

The original datasets are presented in the article and Supplementary Materials. Further inquiries can be directed to the corresponding author.

## Supplementary Materials

The following supporting information can be downloaded at: https://www.mdpi.com/article/10.3390/ijms25158532/s1.

## Abbreviations

Acute Myeloid Leukemia: AML; FMS-related receptor tyrosine kinase 3: FLT3; Tyrosine Kinase Inhibitors: TKIs; Gilteritinib: GILT; Oligomycin: OA; mDNA: mitochondrial genome; nDNA: nuclear genome; NFκB2: Nuclear Factor-kappa B subunit 2; TFAM: mitochondrial transcription factor A; TFB2M: mitochondrial transcription factor B2; POLRMT: mitochondrial RNA polymerase; LSP: light strand promoter; HSPs: heavy strand promoters; FC: flow cytometry; mPTP: mitochondrial permeability transition pores.
