# Supplementary material for: Discovery of NFκB2-Coordinated Dual Regulation of Mitochondrial and Nuclear Genomes Leads to an Effective Therapy for Acute Myeloid Leukemia
Source: Int J Mol Sci. 2024 Aug 5;25(15):8532. doi: 10.3390/ijms25158532 (PMC11313218; doi:10.3390/ijms25158532)
Supplement: Supplementary file 1 [file ijms-25-08532-s001.zip › ijms-3125416-supplementary.pdf]

International Journal of *Molecular Sciences*

MDPI

Article

Discovery of NFκB2-Coordinated Dual Regulation of Mitochondrial and Nuclear Genomes Leads to an Effective Therapy for Acute Myeloid Leukemia

Yi Xu [1,2,3,*], David J. Baylink [2], Jeffrey Xiao [2], Lily Tran [2], Vinh Nguyen [2], Brandon Park [2], Ismael Valladares [2], Scott Lee [4], Kevin Codorniz [4], Laren Tan [5], Chien-Shing Chen [1,3], Hisham Abdel-Azim [1,6,7], Mark E. Reeves [1,3], Hamid Mirshahidi [1,3], Guido Marcucci [8], Huynh Cao [1,3]

[1]Division of Hematology and Oncology, Department of Medicine, School of Medicine, Loma Linda University, Loma Linda, CA 92354, USA.

[2]Division Regenerative Medicine, Department of Medicine, School of Medicine, Loma Linda University, Loma Linda, California, CA 92354, USA.

[3]Cancer Center, Loma Linda University, Loma Linda, California, CA 92354, USA.

[4]Division of Endocrinology, Diabetes & Metabolism, Department of Medicine, School of Medicine, Loma Linda University, Loma Linda, California, CA 92354, USA.

[5]Division of Pulmonary, Critical Care, Hyperbaric and Sleep Medicine, Department of Medicine, School of Medicine, Loma Linda University, Loma Linda, California, CA 92354, USA.

[6]Division of Transplant and Cell Therapy, Loma Linda University Cancer Center; Loma Linda, California, CA 92354, USA.

[7]Division of Hematology and Oncology, Department of pediatrics, Loma Linda University, Loma Linda, California, CA 92354, USA.

[8]Department of Hematological Malignancies Translational Science, Gehr Family Center for Leukemia Research, City of Hope Medical Center and Beckman Research Institute, Duarte, California, CA 91010, USA

* Correspondence: dyxu@llu.edu; Tel.: +1-9096515887

Supplementary Materials:

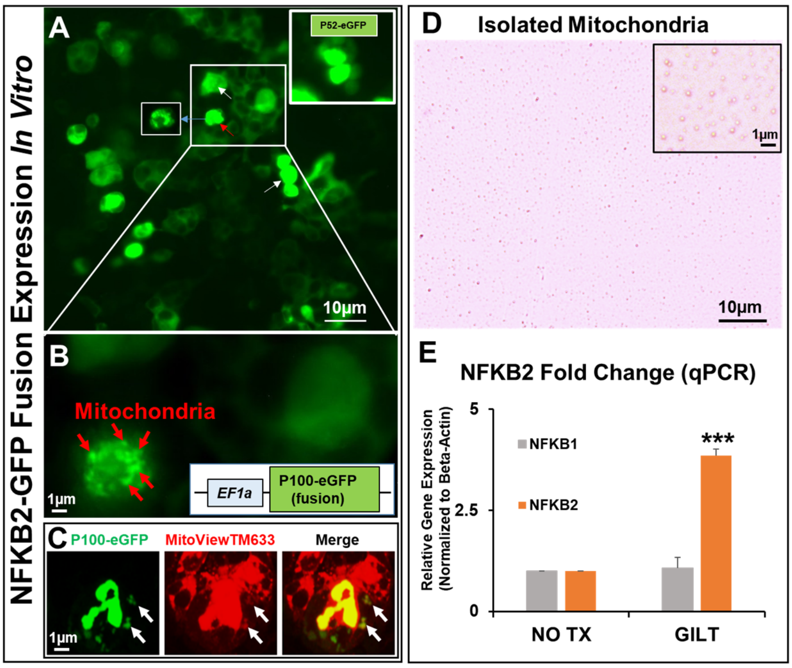

Supplementary Figure S1: NFκB2 was genetically localized in human mitochondria. (N=3)

A) Lentiviral constructs containing transgenes of *P100-eGFP*-fusion (full length *NFκB2*) or *P52-eGFP*-fusion (truncated *NFκB2*) were generated and transfected into HEK-293T cells, a human cancer cell line. P100-eGFP was localized in cytoplasm, while P52-eGFP was localized in nuclei (Inset); When the focus of the microscope was adjusted, the cell (indicated by red arrow) could be observed with multiple GFP+ mitochondria in this cell at low magnification (indicated by blue arrow); There are multiple cells containing visible GFP+ mitochondria at low magnification with a scale bar of 10μm (indicated by white arrows);
B) Higher magnification of ***A)*** revealed many GFP+ mitochondria (indicated by red arrows) in this P100-eGFP+ cell with a scale bar of 1μm;
C) Representative fluorescent images of co-localization of MitoView™633-stained mitochondria and GFP+ mitochondria (indicated by white arrows) with a scale bar of 1μm;
D) Representative phase-bright image of mitochondria isolated from MV4-11 cells; Inset: high magnification image of isolated mitochondria;
E) Gene expressions of *NFκB1* and *NFκB2* in isolated mitochondria from MV4-11 cells treated with or without GILT, analyzed by qPCR. Data of mRNA expressions show the fold change (normalized to *β-actin*) of *NFκB1* and *NFκB2* in different treatment groups;
Where applicable, data are means ± SEM. ***P<0.005, N=3.

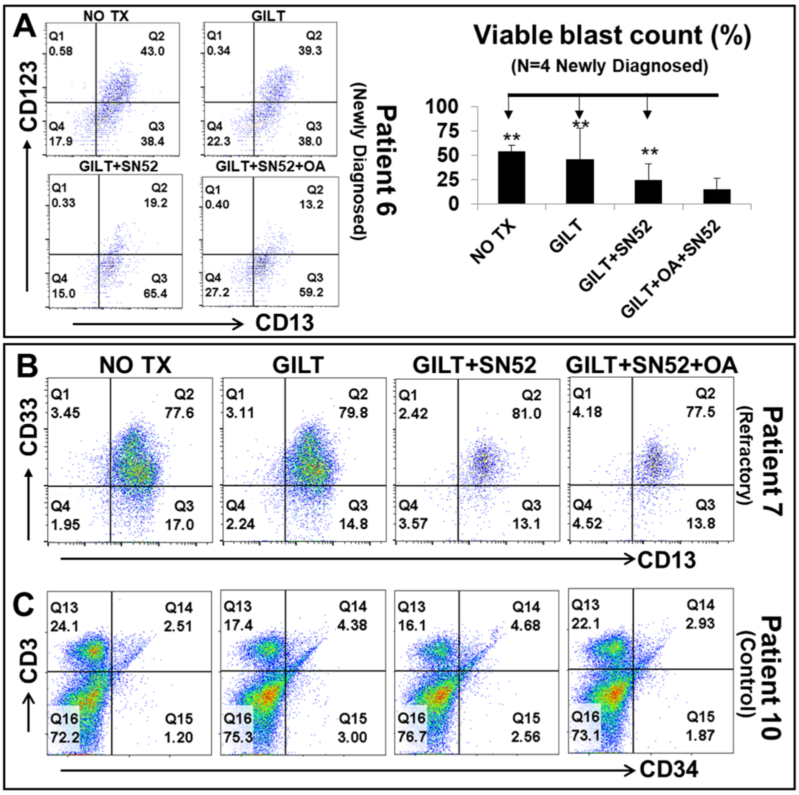

Supplementary Figure S2: The triplet therapy displayed potent anti-leukemia effect on *FLT3*-mut blasts of newly diagnosed and refractory AML patients *ex vivo*.
A) Representative FC plots of viable blasts (gated on viability dye negative populations) from different experimental groups of bone marrow mononuclear cells (BMMNC) from a newly diagnosed AML patient 6 with NO TX, 80nM GILT, 80nM GILT+15μM SN52, and 80nM GILT+15μM SN52+100nM OA, which were analyzed by CD13 and CD123 according

to patient 6's clinical profile; **Right table:** Cumulative percentage data of viable blasts in different treatment groups of four newly diagnosed AML patients 1-3 and patient 6; Where applicable, data are means ± SEM. **P<0.01.

B) Representative FC plots of viable blasts (gated on viability dye negative populations) from different experimental groups of bone marrow mononuclear cells (BMMNC) from a refractory AML patient 7 with NO TX, 80nM GILT, 80nM GILT+15μM SN52, and 80nM GILT+15μM SN52+100nM OA, which were analyzed by CD13 and CD33 according to patient 7's clinical profile;

C) Representative FC plots of viable cells (gated on viability dye negative populations) from different experimental groups of the peripheral blood (PB) specimen from a healthy patient 10 with NO TX, 80nM GILT, 80nM GILT+15μM SN52, and 80nM GILT+15μM SN52+100nM OA, which were analyzed by CD3 and CD34.

Supplementary Table S1: List of Reagents used in this study.

**List of Reagents**				
**Antibody/Reagents**	**Abbreviation/Name in the text**	**Cat. #**	**Company**	**Species Reactivity**
**MitoView™633**		70055	Biotium	
**MEBCYTO Apoptosis Kit (Annexin V-FITC Kit)**		4700	MBL	
**ATP Assay Kit**		Ab83355	Abcam	
**Viability Dye eFluor™ 780**	Viability Dye	65-0865-14	eBioscience	
**CD3-PE/Cyanine7**	CD3	300420	Biolegend	Human
**CD13-PE-Cyanine7**	CD13	301712	Biolegend	Human
**CD33-APC**	CD33	303408	Biolegend	Human
**CD33-FITC**	CD33	366620	Biolegend	Human
**CD34-APC**	CD34	343510	Biolegend	Human
**CD34-PE**	CD34	348057	BD Biosciences	Human
**CD34-PERCP**	CD34	340666	BD Biosciences	Human
**CD44-PE/Cyanine7**	CD44	338816	Biolegend	Human
**CD117-APC**	CD117 (c-kit)	313206	Biolegend	Human
**CD123-FITC**	CD123	306013	Biolegend	Human
**TFAM**		**MA5-16148**	ThermoFisher Sci	Human
**TFB2M**		PA5-40897	ThermoFisher Sci	Human
**MT-CO1-FITC**	COX1	Orb43663	Biorbyt	Human
**NRF1-PE**		Orb495416	Biorbyt	Human
**PE anti-mouse IgG**		406607	Biolegend	Mouse
**APC anti-mouse IgG**		405308	Biolegend	Mouse
**PE anti-rabbit IgG**		406421	Biolegend	rabbit
**Gilteritinib (ASP2215)**	GILT	S7754	SELLECKCHEM	

**Oligomycin**	OA	HY-N6782	MedChemExpress	
**SN52**		HY-P3229	MedChemExpress	

Supplementary Table S2: List of Primers (*OriGene) used in this study.

#	Name (HUMAN)	Forward Sequence	Reverse Sequence
1	***NFκB1****	GCAGCACTACTTCTTGACCACC	TCTGCTCCTGAGCATTGACGTC
2	***NFκB2****	GGCAGACCAGTGTCATTGAGCA	CAGCAGAAAGCTCACCACACTC
3	***TFAM****	GTGGTTTTCATCTGTCTTGGCAAG	TTCCCTCCAACGCTGGGCAATT
4	***GDF15****	CAACCAGAGCTGGGAAGATTCG	CCCGAGAGATACGCAGGTGCA
5	***NRF1****	GGCAACAGTAGCCACATTGGCT	GTCGTCTGGATGGTCATCTCAC
6	***MT-CO1****	TCTCAGGCTACACCCTAGACCA	ATCGGGGTAGTCCGAGTAACGT
9	***β-Actin****	CACCATTGGCAATGAGCGGTTC	AGGTCTTTGCGGATGTCCACGT

Supplementary Table S3: A list of transgenic cell lines of *NFκB2* overexpression, *NFκB2-eGFP* fusion reporters and shRNA-*NFκB2* knockdown that were newly generated for this study. ORF: open reading frame; CS: custom-built lentivector.

Cell lines with lentiviral overexpression	Gene name (ORF, NCBI ID)	Catalog # (GeneCopoeia)	Promoters for ORF	Fluorescent reporter
***NFKB2-eGFP*-MV4-11**	*NFKB2* (NM_001077494.3)	EX-Z4293-Lv225	EF1a	GFP
***NFKB1-mCherry*-MV4-11**	*NFKB* (NM_003998.3)	EX-F0208-Lv224	EF1a	mCherry
***RELB-mCherry*-MV4-11**	*RELB* (NM_006509.3)	EX-G0029-Lv224	EF1a	mCherry
***NFKB2-RELB-eGFP*-MV4-11**	*NFKB2; RELB*	**CS**-Z4293-Lv225-01	EF1a (NFKB2); IRES (RELB)	GFP
***NFKB2-eGFP/RELB-mCherry*-MV4-11**	*NFKB2; RELB*	EX-Z4293-Lv225; EX-G0029-Lv224	EF1a	GFP; mCherry
***NFKB2-eGFP/NFKB1-mCherry*-MV4-11**	*NFKB2, NFKB1*	EX-Z4293-Lv225; EX-F0208-Lv224	EF1a	GFP; mCherry
***GFP*-MV4-11**	NONE	EX-NEG-Lv225	IRES2	GFP
**Cell lines with NFKB2-fusion reporter**	**Gene name**	**Catalog # (GeneCopoeia)**	**Promoters**	**Fluorescent reporter**
***P100-eGFP*-HEK293T**	*NFKB2 (P100)-eGFP* fusion	**CS**-Z4293-Lv224-01	EF1a; IRES2	GFP; mCherry
***P52-eGFP*-HEK293T**	*NFKB2 (P52)-eGFP* fusion	**CS**-Z4293-Lv224-02	EF1a; IRES2	GFP; mCherry
***P100-eGFP*-MV4-11**	*NFKB2 (P100)-eGFP* fusion	**CS**-Z4293-Lv224-03	EF1a	GFP
**Knockdown cell lines**	**Name of gene targets**	**Catalog # (OriGene)**	**Promoters**	**Fluorescent reporter**
**ShRNA-Scramble-MV4-11**	NONE	TL311187V	U6	GFP
**ShRNA-*NFKB2*(A)-MV4-11**	*NFKB2*	TL311187V	U6	GFP
**ShRNA-*NFKB2*(B)-MV4-11**	*NFKB2*	TL311187V	U6	GFP
**ShRNA-*NFKB2*(C)-MV4-11**	*NFKB2*	TL311187V	U6	GFP
**ShRNA-*NFKB2*(D)-MV4-11**	*NFKB2*	TL311187V	U6	GFP

Supplementary Table S4: A list of transgenic cell lines of mitochondrial and nuclear promoter assays that were newly generated for this study. CS: custom-built lentivector.

Promoter assay cell lines	Name of promoters	Catalog # (GeneCopoeia)	Host cell lines	Reporter
**HSP1/2-*NFKB2-eGFP*-MV4-11**	HSP1/2 (lentivector construct 1, **Fig.4**)	**CS**-HPRM2197L-LvPG04-01	***NFKB2-eGFP*-MV4-11**	Gaussia Luciferase
**HSP1/2-*eGFP*-MV4-11**	HSP1/2 (lentivector construct 1, **Fig.4**)	**CS**-HPRM2197L-LvPG04-01	***GFP*-MV4-11**	Gaussia Luciferase
**LSP-*NFKB2-eGFP*-MV4-11**	LSP (lentivector construct 2, **Fig.4**)	**CS**-HPRM2198L-LvPG04-01	***NFKB2-eGFP*-MV4-11**	Gaussia Luciferase
**LSP-*eGFP*-MV4-11**	LSP (lentivector construct 2, **Fig.4**)	**CS**-HPRM2198L-LvPG04-01	***GFP*-MV4-11**	Gaussia Luciferase
**Enhancer-*NFKB2-eGFP*-MV411**	Enhancer (lentivector construct 3, **Fig.4**)	**CS**-HPRM2219L-LvPG04-01	***NFKB2-eGFP*-MV4-11**	Gaussia Luciferase
**Enhancer-*eGFP*-MV4-11**	Enhancer (lentivector construct 3, **Fig.4**)	**CS**-HPRM2219L-LvPG04-01	***GFP*-MV4-11**	Gaussia Luciferase
**HSP1-*NFKB2-eGFP*-MV4-11**	Enhancer/HSP1 (lentivector construct 4, **Fig.4**)	**CS**-HPRM2220L-LvPG04-01	***NFKB2-eGFP*-MV4-11**	Gaussia Luciferase
**HSP1-*eGFP*-MV4-11**	Enhancer/HSP1 (lentivector construct 4, **Fig.4**)	**CS**-HPRM2220L-LvPG04-01	***GFP*-MV4-11**	Gaussia Luciferase
**TFAM-*NFKB2-eGFP*-MV4-11**	*TFAM*	HPRM61407-LvPG04	***NFKB2-eGFP*-MV4-11**	Gaussia Luciferase
**TFAM-*eGFP*-MV4-11**	*TFAM*	HPRM61407-LvPG04	***GFP*-MV4-11**	Gaussia Luciferase
